# Tau-targeting antisense oligonucleotide MAPT_Rx_ in mild Alzheimer’s disease: a phase 1b, randomized, placebo-controlled trial

**DOI:** 10.1038/s41591-023-02326-3

**Published:** 2023-04-24

**Authors:** Catherine J. Mummery, Anne Börjesson-Hanson, Daniel J. Blackburn, Everard G. B. Vijverberg, Peter Paul De Deyn, Simon Ducharme, Michael Jonsson, Anja Schneider, Juha O. Rinne, Albert C. Ludolph, Ralf Bodenschatz, Holly Kordasiewicz, Eric E. Swayze, Bethany Fitzsimmons, Laurence Mignon, Katrina M. Moore, Chris Yun, Tiffany Baumann, Dan Li, Daniel A. Norris, Rebecca Crean, Danielle L. Graham, Ellen Huang, Elena Ratti, C. Frank Bennett, Candice Junge, Roger M. Lane

**Affiliations:** 1 Dementia Research Centre, National Hospital for Neurology and Neurosurgery, University College London, London, UK; 2 Karolinska University Hospital, ME Aging, Stockholm, Sweden; 3 Sheffield Teaching Hospital NHS Foundation Trust, NIHR Sheffield Clinical Research Facility and NIHR Sheffield Biomedical Research Centre, Royal Hallamshire Hospital, Sheffield, UK; 4 Alzheimer Center Amsterdam, Department of Neurology, Amsterdam Neuroscience, Vrije Universiteit Amsterdam, Amsterdam UMC, Amsterdam, the Netherlands; 5 University Medical Center Groningen / RUG, Alzheimer Center Groningen, Groningen, the Netherlands; 6 Douglas Mental Health University Institute and McConnell Brain Imaging Centre of the Montreal Neurological Institute, McGill University, Montreal, Quebec Canada; 7 Memory Clinic, Psychiatry - Cognition and Geriatric Psychiatry, Sahlgrenska University Hospital, Gothenburg/Molndal, Sweden; 8 German Center for Neurodegenerative Diseases, DZNE, and Department of Neurodegenerative Diseases and Geriatric Psychiatry, University Hospital Bonn, Bonn, Germany; 9 CRST Oy; Turku PET Centre University of Turku and Turku University Hospital, Turku, Finland; 10 Department of Neurology University of Ulm and DZNE, Ulm, Germany; 11 Pharmakologisches Studienzentrum Chemnitz GmbH Mittweida, Mittweida, Germany; 12 Ionis Pharmaceuticals, Carlsbad, CA USA; 13 Biogen, Cambridge, MA USA

**Keywords:** Alzheimer's disease, Drug development

## Abstract

Tau plays a key role in Alzheimer’s disease (AD) pathophysiology, and accumulating evidence suggests that lowering tau may reduce this pathology. We sought to inhibit *MAPT* expression with a tau-targeting antisense oligonucleotide (MAPT_Rx_) and reduce tau levels in patients with mild AD. A randomized, double-blind, placebo-controlled, multiple-ascending dose phase 1b trial evaluated the safety, pharmacokinetics and target engagement of MAPT_Rx_. Four ascending dose cohorts were enrolled sequentially and randomized 3:1 to intrathecal bolus administrations of MAPT_Rx_ or placebo every 4 or 12 weeks during the 13-week treatment period, followed by a 23 week post-treatment period. The primary endpoint was safety. The secondary endpoint was MAPT_Rx_ pharmacokinetics in cerebrospinal fluid (CSF). The prespecified key exploratory outcome was CSF total-tau protein concentration. Forty-six patients enrolled in the trial, of whom 34 were randomized to MAPT_Rx_ and 12 to placebo. Adverse events were reported in 94% of MAPT_Rx_-treated patients and 75% of placebo-treated patients; all were mild or moderate. No serious adverse events were reported in MAPT_Rx_-treated patients. Dose-dependent reduction in the CSF total-tau concentration was observed with greater than 50% mean reduction from baseline at 24 weeks post-last dose in the 60 mg (four doses) and 115 mg (two doses) MAPT_Rx_ groups. Clinicaltrials.gov registration number: NCT03186989.

## Abstract (web summary)

Evaluation of a tau-targeting antisense oligonucleotide in a phase 1 trial of patients with mild AD found it was well tolerated and resulted in a sustained reduction of tau protein levels.

## Main

Alzheimer’s disease (AD) is a progressive neurodegenerative disorder characterized by cognitive and functional decline resulting in substantial disability^1^. Onset of pathology is marked by progression of neuroimaging and fluid biomarker measures (preclinical phase) before the appearance of subtle cognitive changes, known as mild cognitive impairment (MCI). The eventual progression to dementia occurs over a variable period of time and is characterized by cognitive and behavioral symptoms that impair an individual’s ability to function in daily life^2^. Symptom onset typically occurs in patients aged 65 years and older, while symptom onset before age 65 accounts for less than 5% of all patients with AD^3^. Historically, diagnosis of AD has been primarily focused on clinical criteria^4^, but accumulating evidence has demonstrated that cerebrospinal fluid (CSF) biomarkers, including amyloid-β 42 (Aβ42) and tau (total tau (t-tau) and phosphorylated tau181 (p-tau181)) as well as positron emission tomography (PET)-amyloid and PET-tau, are reliable surrogate measures of neuropathologic change enabling more robust characterization of patients across the AD continuum^5–7^. For most patients with AD, treatment remains limited to multidisciplinary management of symptoms, including pharmacological therapies that have no disease-modifying impact. The recent US Food and Drug Administration accelerated approvals of aducanumab and lecanemab provide the first treatment targeting a key disease mechanism in AD, the accumulation of amyloid plaques, for patients with MCI or mild AD. There are over 50 million people worldwide currently living with dementia mostly due to AD, and this number is expected to double every 20 years^8^; therefore, additional disease-modifying treatments to prevent or slow progression of this disease remain a significant unmet need.

Growing evidence suggests that aggregated, hyperphosphorylated tau may be a key driver of neurodegeneration in AD. Tau protein is encoded by the microtubule-associated protein tau (*MAPT*) gene and is a microtubule-associated protein primarily expressed in neurons^9^. Under pathogenic conditions, hyperphosphorylated tau accumulates intracellularly, aggregating into oligomers and fibrils resulting in intraneuronal neurofibrillary tangles, and is associated with cognitive decline in AD^10,11^. Tau is also secreted from neurons, spreading through specific neural networks via a trans-synaptic route causing propagation of tau pathology that is associated with further synaptic dysfunction and neuronal loss^12–15^. Preclinical evidence has demonstrated that tau reduction prevents specific Aβ-mediated deficits, supporting a central role of tau in mediating Aβ toxicity in the early pathogenesis of AD. Intracerebroventricular injection of purified Aβ oligomers into adult rodents has been shown to impair long-term potentiation, and this impairment of long-term potentiation in hippocampal slices of wild-type mice is prevented in tau knockout mice^16,17^. Evidence from amyloid precursor protein (APP) mouse models of AD have shown that both hetero- and homozygote tau deficiency rescued premature mortality and prevented memory deficits in transgenic mice expressing familial AD mutations in human APP (hAPP), which appeared to be conferred by reduced susceptibility to excitotoxicity in tau knockout mice^18,19^. In addition, knocking out tau rescued memory impairments, loss of synapses and premature death in hAPP mice expressing human mutant PS1 (ref. ^20^). Given the important role of tau in AD pathophysiology and the accumulating evidence that lowering tau may reduce this pathological effect, we sought to inhibit *MAPT* expression and thus reduce tau levels, directly targeting a key disease effector mechanism in patients with AD.

MAPT_Rx_ (ISIS 814907/BIIB080) is an antisense oligonucleotide (ASO) designed to reduce concentrations of *MAPT* messenger RNA. MAPT_Rx_ is a chemically modified synthetic oligomer that is complementary to an 18-nucleotide stretch of *MAPT* pre-mRNA. MAPT_Rx_ binds within intron 9 of the *MAPT* pre-mRNA through Watson–Crick base pairing, with hybridization resulting in endogenous ribonuclease H1-mediated degradation of the *MAPT* mRNA, inhibiting translation of the tau protein. ASO-mediated selective reduction of *MAPT* mRNA leads to lowered tau protein levels and sustained amelioration of disease-associated phenotypes in transgenic animal models of tauopathy and hyperexcitability^21–24^. For example, *MAPT* mRNA-targeting ASOs in a mouse model of tauopathy resulted in a 50% reduction of endogenous intracellular tau, reduced cell-to-cell spread of oligomerized tau, markedly reduced neuronal and cognitive impairments and was not associated with adverse consequences^15^. Knockdown of tau in animal models and primary neurons did not impair microtubule assembly, axonal transport or sensory, motor or cognitive behavior tasks^22,25,26^. Moreover, complete tau knockout mice had normal development and cognition with only a minor motor phenotype developing later in life^27–30^. These data mitigate potential safety concerns of lowering tau as a therapeutic approach for AD and other tauopathies.

In this study, we aimed to evaluate the safety and pharmacokinetics (PK) of MAPT_Rx_ in patients with mild AD and explore the hypothesis that precisely targeted degradation of *MAPT* mRNA using an ASO would result in lowering of t-tau and phosphorylated tau (p-tau) levels in the central nervous system (CNS). We report the results of a first-in-human phase 1b clinical trial evaluating a tau-targeting ASO administered intrathecally as a bolus in adults with mild AD.

## Results

### Patients

**Fig. 1:**
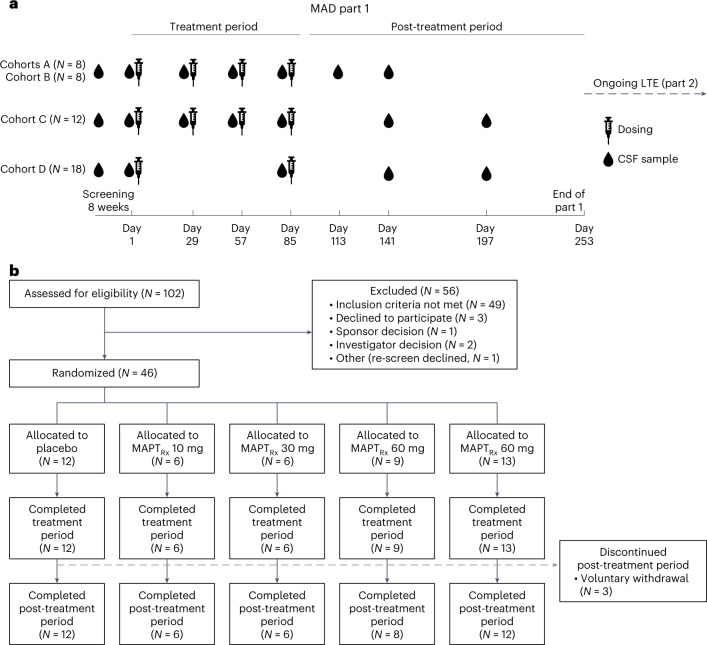
Trial design and patient flow diagram. **a**, Dosing and CSF sample collection for MAD part 1. CSF samples were obtained before the administration of study drug on days 1, 29, 57 and 85 for cohort A (10 mg MAPT_Rx_ or placebo monthly), cohort B (30 mg MAPT_Rx_ or placebo monthly) and cohort C (60 mg MAPT_Rx_ or placebo monthly) and on days 1 and 85 for cohort D (115 mg MAPT_Rx_ or placebo quarterly). The results of CSF samples obtained during screening and on day 1 (baseline) were averaged to serve as the baseline assessment, and the CSF samples on days 29, 57 and 85 served as 28 day, 56 day or 84 day post-dose trough samples. Two CSF samples were obtained in the post-treatment period, on either day 113 or day 141 for cohorts A and B and day 141 and day 197 for cohorts C and D. **b**, Patient flow during MAD part 1. Eligible patients were randomly assigned in a 3:1 ratio to receive the ASO MAPT_Rx_ or placebo in all cohorts.

From August 2017 through February 2020, 102 participants were screened for eligibility and 46 underwent randomization according to the protocol (Fig. 1). All participants received all scheduled doses of the study drug (MAPT_Rx_ or placebo) during multiple ascending dose (MAD) part 1. Three participants (6.5%) voluntarily withdrew from the study during the post-treatment period: one each from the placebo, 60 mg MAPT_Rx_ cohort (four total doses administered monthly) and 115 mg MAPT_Rx_ cohort (two total doses administered quarterly). Participants completing MAD part 1 were eligible to participate in the open-label long-term extension (LTE) part 2. Participants randomized to 10 mg or 30 mg MAPT_Rx_ (four total doses administered monthly) cohorts experienced a variable gap between completion of the 13 week treatment period of the MAD in part 1 and day 1 of LTE part 2 since the protocol was amended to add the LTE after participants in these cohorts had begun the study. Transition to LTE part 2 was seamless after a 23 week post-treatment period for participants in the 60 mg and 115 mg MAPT_Rx_ cohorts.

The characteristics of participants at baseline were representative of relatively younger (mean age of 66 years in both placebo and MAPT_Rx_ groups) patients with mild AD and were generally similar across trial groups (Table 1). MAPT_Rx_ groups and placebo group had similarly elevated CSF levels of mean t-tau (405.6 ± 132.7 and 387.3 ± 120.9 pg ml^−1^, respectively) and p-tau181 (40.7 ± 14.2 and 38.7 ± 13.0 pg ml^−1^, respectively) concentrations at baseline. The mean Clinical Dementia Rating (CDR) Sum of Boxes score at baseline was numerically lower for MAPT_Rx_ 60 mg and 115 mg treatment groups due to an amendment during the study, which allowed inclusion of participants with a CDR Global Score of 0.5 and Memory Score of 1 in addition to participants with a CDR Global Score of 1.0.

**Table 1:** Characteristics of patients at baseline^a^

	Placebo	MAPT_Rx_				
Characteristic	(*N* = 12)	MAPT_Rx_ groups (*N* = 34)	10 mg monthly (*N* = 6)	30 mg monthly (*N* = 6)	60 mg monthly Q4W (*N* = 9)	115 mg quarterly (*N* = 13)
Age, years	66 ± 4.6	66 ± 6.1	64 ± 5.2	65 ± 6.1	66 ± 6.8	67 ± 6.3
Age at diagnosis, years	65 ± 4.6	64 ± 6.4	62 ± 5.2	64 ± 6.9	64 ± 6.6	65 ± 6.9
Female, no. (%)	6 (50)	17 (50)	2 (33)	4 (67)	5 (56)	6 (46)
Race—white, no. (%)	12 (100)	34 (100)	6 (100)	6 (100)	9 (100)	13 (100)
MMSE Total Score	24.2 ± 1.7	23.5 ± 2.4	21.5 ± 1.6	24.5 ± 1.4	24.6 ± 2.5	23.2 ± 2.5
RBANS Total Score	64.9 ± 10.2	68.2 ± 12.1	58.8 ± 11.2	69.2 ± 12.1	69.9 ± 9.1	70.9 ± 13.4
CDR Global Score, no. (%)						
0.5	7 (58)	23 (68)	0 (0)	3 (50)	9 (100)	11 (85)
1	5 (42)	11 (32)	6 (100)	3 (50)	0 (0)	2 (15)
CDR Sum of Boxes	4.1 ± 1.3	3.7 ± 1.1	4.8 ± 0.5	4.7 ± 1.0	2.9 ± 0.6	3.3 ± 1.1
Concomitant medications, no. (%)						
Anticholinesterases	7 (58)	21 (62)	4 (67)	5 (83)	4 (44)	8 (62)
Memantine	1 (8)	7 (21)	2 (33)	0 (0)	3 (33)	2 (15)
Estrogen replacement	0 (0)	3 (9)	1 (17)	0 (0)	1 (11)	1 (8)
APOE4 carrier (%)	8 (67)	25 (74)	5 (83)	3 (50)	6 (67)	11 (85)
Homozygous	2 (16.7)	8 (23.5)	2 (33.3)	0 (0)	3 (33.3)	3 (23.1)
Heterozygous	6 (50)	17 (50)	3 (50)	3 (50)	3 (33.3)	8 (61.5)
CSF t-tau (λpg ml^−1^)	387.3 ± 120.9	405.6 ± 132.7	364.6 ± 98.1	386.4 ± 152.3	391.0 ± 111.8	443.4 ± 153.8
p-tau181 (pg ml^−1^)	38.7 ± 13.0	40.7 ± 14.2	39.1 ± 13.0	38.6 ± 16.6	39.5 ± 12.6	43.2 ± 15.9
t-tau/Aβ42	0.6 ± 0.2	0.6 ± 0.2	0.6 ± 0.2	0.6 ± 0.1	0.5 ± 0.1	0.6 ± 0.2

^a^Plus–minus values are mean ± standard deviation. Patients were assigned to receive either placebo or ascending doses of the ASO MAPT_Rx_. Percentages may not total 100 because of rounding. RBANS, Repeatable Battery for the Assessment of Neuropsychological Status; APOE4, apolipoprotein epsilon 4.

### Primary endpoint of safety

Adverse events (AEs) were reported in 94% of participants treated with MAPT_Rx_ and 75% of participants treated with placebo; all events were considered mild (88%) or moderate (12%) in severity by investigators (Table 2; for AE incidence and frequency by treatment group, see Extended Data Table 1). A greater percentage of participants receiving MAPT_Rx_ experienced mild AEs compared with those receiving placebo; the incidence of moderate AEs was similar.

**Table 2:** AEs reported in at least three patients receiving MAPT_Rx_ according to severity^a^

Event	Mild (grade 1)		Moderate (grade 2)		Severe (grade 3)	
	MAPT_Rx_ groups (*N* = 34)	Placebo group (*N* = 12)	MAPT_Rx_ groups (*N* = 34)	Placebo group (*N* = 12)	MAPT_Rx_ groups (*N* = 34)	Placebo group (*N* = 12)
Number of patients with event (%)						
Any AE (%)	21 (62)	5 (42)	11 (32)	4 (33)	0	0
Any serious AE	0	0	0	2 (16.7)	0	0
Post-LP headache^b^	13 (38)	1 (8)	2 (6)	3 (25)	0	0
Procedural pain	4 (12)	1 (8)	3 (9)	0	0	0
Musculoskeletal pain	3 (9)	0	1 (3)	0	0	0
Vomiting	4 (12)	0	0	0	0	0
Back pain	2 (6)	1 (8)	1 (3)	0	0	0
Confusional state	2 (6)	0	1 (3)	0	0	0
Contusion	1 (3)	0	2 (6)	0	0	0
Diarrhea	2 (6)	0	1 (3)	0	0	0
Dizziness	3 (9)	1 (8)	0	0	0	0
Fatigue	3 (9)	0	0	0	0	0
Myalgia	2 (6)	1 (8)	1 (3)	0	0	0
Nasopharyngitis	3 (9)	2 (17)	0	0	0	0
Nausea	3 (9)	0	0	0	0	0
Tinnitus	3 (9)	0	0	0	0	0

^a^Shown are AEs that occurred from the first dose of study drug through the end of MAD part 1 (treatment and post-treatment periods). Each AE was rated as mild, moderate or severe, corresponding to grades of 1, 2 and 3, respectively. In addition, serious AEs were rated as life-threatening (grade 4) or not life-threatening. At each level of summation (overall and according to system organ class or preferred term), patients for whom more than one AE was reported were counted only once for the incidence according to the most severe grade, and if there was a missing severity for the same subject, then the non-missing severity, if available, was chosen for the same subject.

^b^Post-LP headache indicates both post-LP syndrome and headache that were potentially related to study LP procedure. Related was defined as ‘related’, ‘possible’ or missing relationship to LP procedure.

The most reported AE in participants treated with MAPT_Rx_ was post-lumbar puncture (LP) headache, which was generally considered mild (*N* = 13); two participants reported post-LP headache considered moderate. Post-LP headache considered potentially related to study procedure occurred after 20% of LPs. There was no evidence of an increased risk of post-LP headache with successive LPs. All post-LP headaches resolved (median duration, <1 day), and no blood patches were required to resolve.

AEs considered potentially related to study drug by Investigators were reported in 15 participants (44%) treated with MAPT_Rx_ and no participants treated with placebo. Investigators were blinded to treatment assignment. Most participants (80%) with AEs considered potentially related to study drug experienced mild AEs. Safety magnetic resonance imaging (MRI) was performed 6 months post-baseline, and no clinically meaningful changes were observed on qualitative neuroradiological review. There were no deaths, dose-limiting AEs or discontinuations of dosing regimens during the trial. One participant experienced a delay of approximately 2 months in study drug administration due to coronavirus disease 2019 restrictions.

Two serious AEs occurred in two participants receiving placebo: hospitalization due to diverticulitis and an emergency room visit due to a minor stroke from which both participants recovered. Neither suicidal behavior nor serious suicidal ideation emerged in any participant during the trial. A mildly increased CSF leukocyte count (26–28 cells mm^−3^, >90% lymphocytes) without any associated symptoms was observed in one participant, a 64-year-old female, 16 weeks after administration of the second MAPT_Rx_ 115 mg dose; MRI with contrast and electroencephalographic results were normal. The participant did not transition to the LTE, but follow-up safety MRI and CSF collection performed post-MAD part 1 completion showed that the pleocytosis had completely resolved (5 months after initial finding). Two participants receiving quarterly 115 mg MAPT_Rx_ experienced mild confusional state and restlessness 1–2 days after their first and second doses, which resolved within 2–4 days of onset. Both participants had a medical history of anxiety and were treated with psychotropic medications before their enrollment in the study.

### Secondary endpoint

**Fig. 2:**
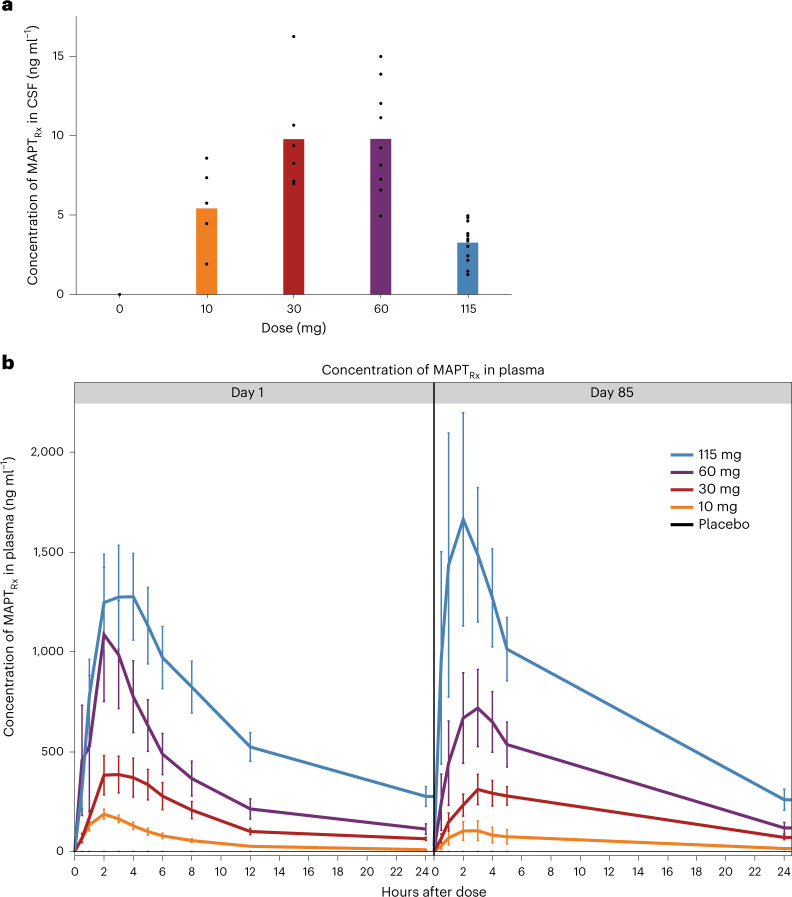
MAPT_Rx_ exposure in CSF and plasma. **a**, The maximum pre-dose CSF concentration of MAPT_Rx_ according to dose group: placebo or the various MAPT_Rx_ dose groups (that is, day 28 ‘trough’ (pre-dose) for placebo, 10 mg (*n* = 6 patients), 30 mg (*n* = 6 patients) and 60 mg (*n* = 9 patients) monthly groups; day 84 trough for 115 mg (*n* = 12 patients) quarterly dose group). Bar represents mean value and points represent individual values. **b**, Mean ± standard error of the mean concentration of MAPT_Rx_ in plasma, according to dose group, over the 24 h periods after the administration of the first dose (left; day 1) and fourth dose for 10 mg (*n* = 6 patients; all timepoints), 30 mg (*n* = 6 patients (*n* = 5 patients at 4 h and 5 h after dose on day 1 and 3 h after dose on day 85)) and 60 mg (*n* = 9 patients (*n* = 8 patients at 1 h after dose on day 1)) monthly dose groups and second dose for 115 mg (*n* = 13 patients on day 1, *n* = 12 patients on day 85) quarterly dose group (right; day 85). Error bars indicate the standard error.

MAPT_Rx_ was measurable in the CSF in all participants receiving MAPT_Rx_ (Fig. 2a). Pre-dose or trough concentrations increased from the 10 mg monthly dose to those observed at the 30 mg and 60 mg monthly doses. Similar trough CSF concentrations were observed after the 30 and 60 mg monthly doses. It is unclear why there is no apparent difference in MAPT_Rx_ CSF trough concentration between the 30 mg and 60 mg MAPT_Rx_ groups. Only six participants in each group received 30 mg or 60 mg MAPT_Rx_, and CSF is not a well-mixed compartment with variable results observed previously^31^. The lowest mean trough CSF concentrations observed after the initial 115 mg quarterly dose were expected considering the longer time for elimination between MAPT_Rx_ doses and sample collection (84 days versus 28 days). Mean trough MAPT_Rx_ concentrations in CSF generally increased during the treatment period in all dose groups probably due to the slow clearance and long elimination half-life of MAPT_Rx_ relative to the dosing interval. The increase over time was less in the 115 mg quarterly cohort compared with the cohorts dosed every month.

### Exploratory endpoints

#### Plasma concentrations of MAPT_Rx_

The median peak plasma concentrations of MAPT_Rx_ were achieved within 4 h after intrathecal (IT) administration and declined to less than 30% of the peak concentration by 24 h after administration. The concentration of MAPT_Rx_ in plasma increased approximately proportionally to the dose over the explored dose range (Fig. 2b). There was no evidence of accumulation of concentration in plasma 24 h after dose administration over the course of the trial, and there was a minor increase (<20%) in the peak concentration at the 115 mg dose level.

#### Concentration of t-tau in CSF

**Fig. 3:**
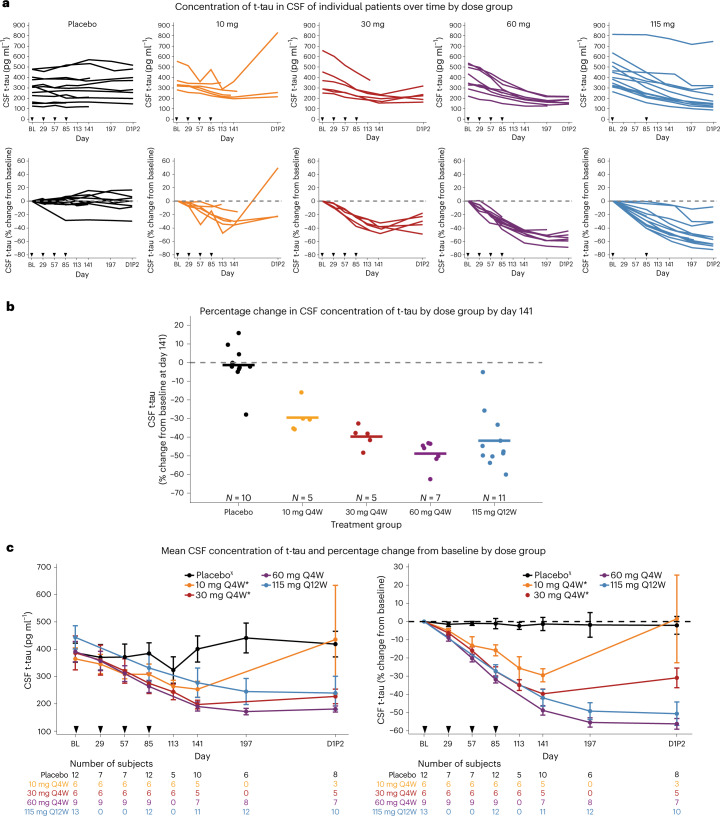
Effect of MAPT_Rx_ on CSF concentrations of t-tau protein. **a**, The concentrations of t-tau in CSF over time for individual patients in each dose group; absolute values, measured in picograms per milliliter (pg ml^−1^), are shown in the top graphs, and the percentage changes from baseline are shown in the bottom graphs. Arrowheads indicate the days on which MAPT_Rx_ or placebo was administered. **b**, The percentage change in the concentration of t-tau in CSF from baseline to the timepoint 56 days after the last dose (day 141). Circles indicate individual patients, and horizontal lines indicate group means. **c**, The mean concentration of t-tau in CSF (left) and the mean percentage change from baseline (right) over time according to dose group. CSF was not collected at 16 weeks post-last dose (day 197) for the 10 mg and 30 mg groups. Error bars indicate the standard error of the mean. Q4W and Q12W indicates dosing every 4 or 12 weeks, respectively. *Participants assigned to cohort A or B did not seamlessly transition to LTE part 2 and experienced a variable gap ranging from 5 to 19 months between completion of MAD part 1 at day 253 and start of LTE part 2 (D1P2). ^χ^Placebo group was pooled. Subjects assigned to cohorts A or B and randomized to placebo had a variable gap between completion of MAD part 1 and start of LTE part 2 (D1P2).

In participants receiving MAPT_Rx_, there were dose-dependent decreases in the concentration of t-tau in CSF. Steady-state maximal reduction of the concentration of CSF t-tau was not reached during the 13 week treatment period, and t-tau concentrations continued to decline during the post-treatment period (Fig. 3a). The mean percentage change from baseline in t-tau concentration at 8 weeks post-last dose was −30%, −40%, −49% and −42% in MAPT_Rx_ 10 mg, 30 mg and 60 mg monthly and 115 mg quarterly groups, respectively (Fig. 3b). In the higher-dose groups with seamless entry into the LTE, CSF t-tau continued to decline at 24 weeks (day 1 LTE part 2) post-last dose in the 60 mg monthly (*N* = 7) and 115 mg quarterly (*N* = 10) MAPT_Rx_ groups (−56% and −51% mean percentage change from baseline, respectively; Fig. 3c). The 60 mg and 115 mg MAPT_Rx_ groups received almost identical cumulative doses of 240 mg and 230 mg, respectively, over the 13 week treatment period, which may account for the similar t-tau reduction observed in both groups. Participants randomized to 10 mg and 30 mg MAPT_Rx_ monthly groups had a variable gap between completing MAD part 1 and starting LTE part 2; the duration between the last dose in MAD part 1 and CSF collection before the first dose in LTE part 2 ranged from 22 to 28 months for the 10 mg group and from 10 to 16 months for the 30 mg group. Despite the prolonged gap, a durable reduction in t-tau concentration was observed in the 30 mg MAPT_Rx_ group (−31% mean percentage change from baseline; *N* = 5) on day 1 of the LTE. T-tau levels had returned to baseline levels in the 10 mg MAPT_Rx_ group (*N* = 3). In participants receiving placebo, the mean percentage change from baseline ranged from −1% to −2.4% among all post-baseline visits (Fig. 3c).

#### Additional exploratory outcomes

**Fig. 4:**
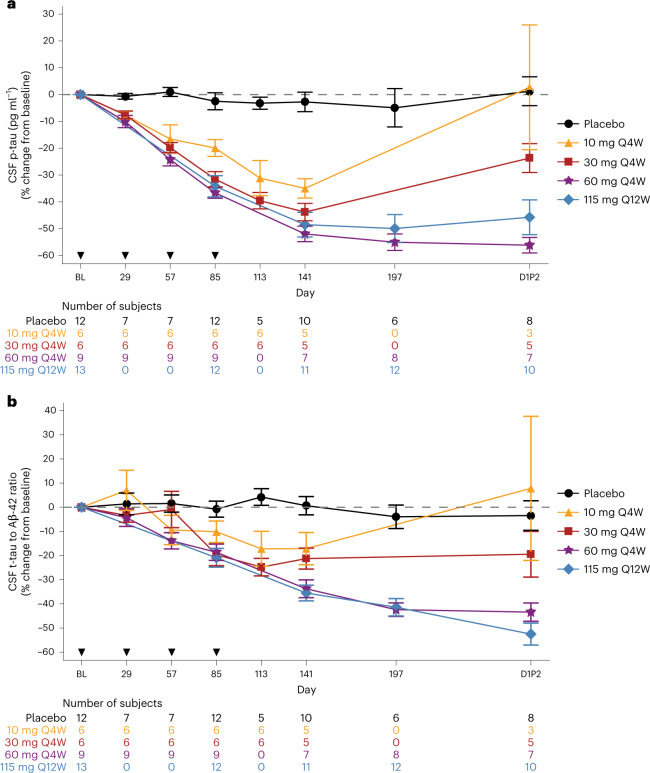
Effect of MAPT_Rx_ on CSF concentrations of p-tau protein and tau/Aβ42. **a**, The mean percentage change from baseline in p-tau over time according to dose group. **b**, The mean percentage change from baseline in the ratio of t-tau to Aβ42 over time according to dose group. Error bars indicate the standard error of the mean. Q4W and Q12W indicates dosing every 4 or 12 weeks, respectively. *Participants assigned to cohort A or B did not seamlessly transition to LTE part 2 and experienced a variable gap ranging from 5 to 19 months between completion of MAD part 1 at day 253 and start of LTE part 2 (D1P2). ^χ^Placebo group was pooled. Subjects assigned to cohorts A or B and randomized to placebo had a variable gap between completion of MAD part 1 and start of LTE part 2 (D1P2).

Reductions similar to those observed for t-tau were observed for p-tau181 concentration and the ratio of t-tau to Aβ42 in CSF (Fig. 4). In participants receiving MAPT_Rx_, there were dose-dependent decreases in the concentration of p-tau181 in CSF 8 weeks post-last dose with mean percentage change from baseline of −35%, −44%, −52% and −49% in MAPT_Rx_ 10 mg, 30 mg and 60 mg monthly and 115 mg quarterly groups, respectively. CSF p-tau181 continued to decline in participants treated with MAPT_Rx_ in 60 mg monthly and 115 mg quarterly groups 24 weeks (day 1 LTE part 2) post-last dose with mean percentage change from baseline of −56% and −46%, respectively.

Performance on functional, cognitive, psychiatric and neurologic clinical outcomes slightly declined as expected for participants with mild AD over the duration of the treatment and post-treatment periods. While no consistent trends were observed across change from baseline on clinical endpoints, further analyses are ongoing to better understand the longitudinal trajectory of the clinical assessments and the relationship with drug exposure and pharmacodynamic (PD) effects. Exploratory CSF parameters including neurofilament light (NfL) and heavy (NfH), neurogranin (NRGN) and YKL-40 showed no dose-responsive effects at 8 weeks post-last dose of MAPT_Rx_ (Supplementary Table 1). CSF NfL levels decreased from baseline in the placebo and 10 mg MAPT_Rx_ groups, and a slight increase from baseline was observed in the 30, 60 and 115 mg MAPT_Rx_ groups. All groups experienced a slight increase from baseline in CSF NfH with the 30 mg MAPT_Rx_ group experiencing the greatest increases in both NfL and NfH. Decrease from baseline in CSF YKL40 was observed in all MAPT_Rx_ treatment groups, whereas no change from baseline was observed in the placebo group. CSF NRGN levels decreased from baseline in the 10, 30 and 60 mg MAPT_Rx_ groups, and no change from baseline was observed in the placebo or 115 mg MAPT_Rx_ groups.

The mean change from baseline in ventricular volume (VV) as a percentage of total intracranial volume 6 months post-baseline was greater in the 10 mg, 30 mg, 60 mg and 115 mg MAPT_Rx_ dose groups (0.5%, 0.7%, 0.7% and 0.6%, respectively) than that observed in the placebo group (0.2%; Extended Data Table 2). Ventricular enlargement (VE) was not observed in qualitative neuroradiological review of safety MRIs from participants in MAPT_Rx_ or placebo groups. Clinical findings potentially associated with VE were not observed during the treatment or post-treatment periods. Whole-brain volume declined slightly from baseline in all groups and did not differ between participants who received placebo and those who received MAPT_Rx._

## Discussion

In this first in human phase 1b study, bolus IT administrations of four monthly doses of MAPT_Rx_ at 10 mg, 30 mg and 60 mg or two quarterly doses at 115 mg to adults with mild AD were not accompanied by any severe or serious AEs during the 13 week treatment period or 23 week post-treatment period. The proportion of participants experiencing AEs was greater in those receiving MAPT_Rx_ versus placebo (94% versus 75%, respectively), and this was mainly due to an increased incidence of mild AEs in the MAPT_Rx_ treatment group (62% versus 42% in placebo). AEs considered potentially related to study drug by investigators were reported more frequently in with MAPT_Rx_-treated participants compared with placebo-treated participants (15 (44%) versus 0, respectively). The most reported AE in participants receiving MAPT_Rx_ was post-LP headache, which was generally mild in severity. All study participants were white, and it will be important in larger, later-phase clinical studies of MAPT_Rx_ to include a diverse patient population to adequately evaluate both efficacy and safety. Overall MAPT_Rx_ treatment was generally well tolerated, with all participants completing the treatment period and over 90% of participants completing the post-treatment period.

MAPT_Rx_ administration resulted in dose- and time-dependent reduction in the concentration of CSF t-tau and p-tau181 with approximately 50% mean reduction from baseline observed 24 weeks post-last dose. Further characterization of MAPT_Rx_ PK and PD data from the LTE will be important for selection of the optimal dose level and frequency for future clinical studies. It is not feasible to directly quantify the reduction of *MAPT* mRNA or tau protein in cortical tissue in a clinical trial; however, it is possible to index PD activity with CSF protein assays^31,32^. Tau is a long-lived protein in the CNS, and thus CSF tau in this study will be a lagging indicator of the reduction of *MAPT* mRNA and newly synthesized tau in the CNS^33^. The predicted MAPT_Rx_ concentrations in brain tissue at all dose levels in this study are sufficient to provide >50% reduction in t-tau production in the cerebral cortex^31^. Therefore, it is not surprising that all doses assessed in this study demonstrated a similar trajectory of CSF tau lowering (Figs. 3 and 4), a trajectory that probably reflects potent reductions in new tau synthesis at all doses with a rate-limiting condition of the elimination of existing tau protein. Many of the tau-targeting antibody and vaccine approaches currently in development aim to reduce spread of specific extracellular tau species and are not predicted to have a major impact on intracellular p-tau^34^. MAPT_Rx_ prevents tau protein production, and should lower the levels of all tau species and subsequent posttranslational modifications with the potential to reduce both pathological spreading of extracellular tau and neuronal dysfunction due to intracellular tau accumulation.

Limitations of evaluating CSF t-tau and p-tau to index target engagement include the clearance of existing tau protein and reliance on tau transport to the CSF. Physiological and pathological conditions may impact the rate of synthesis of tau, passive and active release into the extracellular space, and the clearance of tau including degradation by microglia^33,35,36^. At baseline, CSF tau represents previously synthesized tau. However, at steady state with respect to both ASO distribution and the production and elimination of tau, lowered CSF tau levels should index the reduction of newly synthesized tau^21,22,37^. In the ongoing LTE, steady-state reductions in CSF t-tau protein levels should emerge that reflect the degree to which newly synthesized tau protein is reduced in the CNS. While measuring CSF tau protein can index PD activity, it cannot inform on regional differences in *MAPT* mRNA reduction in CNS tissues.

Quantitative assessment of VV showed greater increases in MAPT_Rx_ treatment groups compared with placebo. Importantly, VE was not evident on qualitative neuroradiological review of safety MRI scans, and there were no clinical correlates. Quantitative increases in VV have been observed in treatment groups relative to placebo in clinical studies of patients with AD^38–41^ while other clinical studies of AD have not reported treatment related- increases^42–45^. The etiology of greater quantitative VE relative to placebo in these studies remains unclear, and associated changes in clinical outcomes were generally not reported in these studies. Slow, progressive whole-brain atrophy (that is, irreversible loss of brain tissue) and VE are characteristic features of AD^46,47^, and neuroinflammation is a known phenomenon in AD^48^. Although ‘pseudoatrophy’ (that is, VE due to resolution of inflammatory edema and gliosis) has been described in clinical studies of multiple sclerosis and AD, it has been challenging to differentiate between treatment-induced pseudoatrophy and disease-related atrophy^38,39,42,49–51^. There were no apparent differences in whole brain volume in MAPT_Rx_ treatment groups versus placebo group in this study. Further work is needed to assess the effect of MAPT_Rx_ treatment on inflammation or gliosis in humans or animal models. Comparing the rate of VE observed in our study with that observed in the Alzheimer’s Disease Neuroimaging Initiative is challenging since our patient population was younger (mean age 66 years versus 75 years in the Alzheimer’s Disease Neuroimaging Initiative)^46,47,52^. Nearly half of the participants in our study were diagnosed before age 65, representing a much higher proportion of participants with early onset AD compared with the general AD population (46% versus 5%). Disease severity, age and genetic status may influence the degree and rate of increase in VV and requires further evaluation in future studies of the drug.

MAPT_Rx_ is the first ASO treatment evaluated in a clinical study of patients with AD. The results from this first in human study demonstrate that MAPT_Rx_ engaged its target, as evidenced by the marked dose-dependent and sustained reductions in the concentration of CSF t-tau, and had an acceptable safety profile in participants with mild AD. Intrathecally administered ASOs have been evaluated in other neurodegenerative diseases including spinal muscular atrophy, Huntington’s disease and amyotrophic lateral sclerosis with mixed results^53–55^. Nusinersen is indicated for the treatment of children and adults with spinal muscular atrophy^56^. While the results of a large phase 3 study of tominersen in patients with more advanced Huntington’s disease did not show evidence of clinical improvement, post hoc exploratory subgroup analysis investigating the association between disease burden and ASO exposure suggests that younger participants with a lower disease burden might derive benefit from less frequent or lower-dose treatment with tominersen in contrast to the other subgroups^57^. The tominersen program continues with a new phase 2 clinical trial exploring different doses of the ASO in younger patients with lower disease burden (EudraCT number 2022-001991-32). Results of a phase 3 study of tofersen treatment in patients with *SOD1* amyotrophic lateral sclerosis did not achieve statistically significant improvements on clinical endpoints, but trends favoring tofersen were observed across clinical outcome measures of respiratory function, muscle strength and quality of life during the 28 week treatment period with further improvement evolving during the open-label extension, including clinical endpoints at week 52. Importantly, robust lowering of CSF SOD1 protein and plasma NfL chains, a marker of axonal injury and neurodegeneration, were observed^53^. Tofersen is currently under US Food and Drug Administration and European Medicines Agency review with approval decisions expected in 2023. Treatment of neurodegenerative diseases with ASOs is still in its infancy, and learnings from recent studies on dose level and frequency as well as trial design (treatment duration, sample size and patient selection) will further improve ASO development and clinical trial designs across neurodegenerative diseases.

This first-in-human study of MAPT_Rx_ has several limitations mainly due to its small size (*N* = 46) as is typical of phase 1 studies. The MAPT_Rx_ doses evaluated in this study achieved the target t-tau reduction of ~50%, but determining whether this reduction is efficacious will require further evaluation in larger, well-controlled trials. Similarly, MAPT_Rx_-treatment related effects on exploratory biomarkers will require further evaluation in larger trials. Changes in NfL, Nfh, YKL40 and NRGN levels did not appear to be dose responsive and were not concordant, but interpretation of the clinical meaningfulness of these results is limited due to the combination of assay variability and small sample size. Lastly, this study was conducted in relatively young participants with mild AD, and it will be important that future studies of MAPT_Rx_ evaluate safety and efficacy in an older population, which may be more representative of late-onset AD. A randomized, double-blind, placebo-controlled phase 2 study of BIIB080 (MAPT_Rx_) is currently underway and includes patients aged 50–80 years with an estimated enrollment of over 700 participants with MCI due to AD or mild AD (Clinicaltrials.gov registration number NCT05399888).

These results demonstrate that antisense-mediated suppression of tau protein synthesis in the CNS of participants with mild AD is possible and warrant further evaluation of the effect of MAPT_Rx_ on the clinical course of patients with AD and in other tauopathies.

## Methods

### Study design and participants

In this randomized, double-blind, placebo-controlled, multicenter, MAD phase 1b trial with an open-label LTE, we evaluated the safety, PK and target engagement of MAPT_Rx_ in participants with mild AD. This study was divided into two parts: MAD part 1, was completed in September 2020, and LTE part 2 was completed in May 2022. Participants had the option to enroll in the open-label LTE upon completion of MAD part 1.

Eligible participants were between the ages of 50 and 74 years and had mild AD, defined by a CDR^58^ Overall Global Score of 1 or Global Score of 0.5 with a Memory Score of 1, Mini-Mental State Examination score (MMSE^59^) of 20–27 inclusive (scores range from 0 to 30, 20–27 may represent mild AD); CSF pattern of low Aβ42 and elevated t-tau and p-tau consistent with diagnosis of AD; and diagnosis of probable AD based on National Institute of Aging-Alzheimer Association criteria^60^ (for further details, see Supplementary Information).

MAD part 1 was conducted at 12 centers in Canada, Finland, Germany, the United Kingdom, the Netherlands and Sweden. A centralized automated randomization system assigned participants 3:1 to receive bolus IT injections of MAPT_Rx_ or placebo (artificial CSF) within each of four dose cohorts during the 13 week treatment period: cohort A, 10 mg monthly, cohort B 30 mg monthly or cohort C, 60 mg monthly (total of four doses each); or cohort D, 115 mg quarterly (total of two doses). Twenty milliliters of CSF were removed before administration of 20 ml of study drug. There was a 23 week post-treatment period during which no study drug was administered. Participants assigned to cohort A or B did not seamlessly transition to LTE part 2 and experienced a variable gap ranging from 5 to 19 months between completion of MAD part 1 at day 253 and registration for LTE part 2. Participants assigned to cohort C or D seamlessly transitioned to LTE part 2. CSF samples were obtained before each administration of study drug (MAPT_Rx_ or placebo), 4 and 8 weeks post-last dose for cohorts A and B, and 8 and 16 weeks post-last dose for cohorts C and D (Fig. 1). Investigators, participants and the sponsor were blinded to trial-group assignments for the trial duration.

The primary objective was evaluation of the safety of MAPT_Rx_. Safety evaluations included collection of AEs, physical and neurologic examination, Columbia Suicide Severity Rating Scale, laboratory assessments, vital signs, electrocardiograms and safety MRI sequences. At each trial visit, participants were queried for other changes in health status and concomitant medications in an open-ended fashion.

The secondary endpoint was the characterization of the PK of MAPT_Rx_ in CSF.

The key exploratory endpoint was CSF t-tau concentration. Additional exploratory endpoints included characterization of MAPT_Rx_ PK in plasma; exploration of the effects of MAPT_Rx_ on PD biomarkers including CSF concentrations of p-tau, Aβ42 and NfL; and clinical, cognitive and neuroimaging assessments relevant to AD (Supplementary Table 1).

### Study drug

MAPT_Rx_ is a second-generation 2′-O-(2-methoxyethyl) ASO complementary to a nucleotide sequence in the human MAPT pre-mRNA transcript. The sequence of MAPT_Rx_ is (5′ to 3′) ccogttTTCTTACCacocct, where capital letters represent 2′-deoxyribose nucleosides, and small letters 2′-(2-methoxyethyl)ribose nucleosides. Nucleoside linkages represented with a subscript o are phosphodiester, and all others are phosphorothioate. Letters represent adenine, 5-methylcytosine, guanine and thymine nucleobases. Hybridization of MAPT_Rx_ to the cognate pre-mRNA via Watson and Crick base pairing results in ribonuclease H1-mediated degradation of the MAPT pre-mRNA, thus selectively preventing production of the tau protein^61^. Dose selection was guided by a preclinical model in mouse and monkey relating dose level to reduction in MAPT mRNA (model described by Tabrizi et al.^31^).

### Study oversight

The trial was conducted in accordance with the Declaration of Helsinki. The trial protocol and all documentation were approved by the institutional review board or independent ethics committee at each investigational site (for list of ethics committees approving the study, see Supplementary Information). All participants provided written informed consent. The trial was sponsored by Ionis Pharmaceuticals, which provided the study drug (MAPT_Rx_ and placebo). Personnel from Ionis Pharmaceuticals designed the trial in conjunction with collaborators from Biogen, academic investigators and other disease experts. A Formal Safety Monitoring Group, composed of sponsor personnel with medical and clinical trial expertise and independent from the conduct of the study, authorized each dose escalation after unblinded review of safety data. The investigators collected the data, which were held and maintained by the sponsor. Data were analyzed by personnel from the sponsor and were interpreted by all authors. The investigators vouch for the fidelity of the trial to the protocol and protocol amendments. The authors vouch for the completeness and accuracy of the data. The authors and sponsor made the decision to submit the manuscript for publication.

### Measurement of brain volumes

We obtained three-dimensional T1-weighted structural MRI scans of the head and transferred these data, blinded to trial-group status, to an independent image-analysis provider that performed quality control, processing and volumetric analyses according to established methods. Whole-brain and regional volume changes were calculated using a validated pipeline implemented in VivoQuantTM, composed of a preprocessing module and a multi-atlas segmentation module, followed by visual inspection and manual editing if needed^62^.

### Biomarker analysis

CSF from participants was analyzed with the following assays: Elecsys β-Amyloid (1-42) CSF, Elecsys Total-Tau CSF and Elecsys Phospho-Tau (181P) CSF performed at Roche Diagnostics); NfL (Uman), NfH (Protein Simple, ELLA), YKL40 (Protein Simple, ELLA) and NRGN (Euroimmune) at Immunologix.

### Statistical analysis

The primary objective of the trial was the evaluation of the safety of MAPT_Rx_ treatment. Safety data were summarized according to treatment group. Quantitative assessments were summarized using descriptive statistics including number of patients, mean, median, standard deviation, standard error of mean, interquartile range (25th percentile, 75th percentile) and range (minimum, maximum). Qualitative assessments were summarized using frequency counts and percentages. All safety analyses were performed in the safety population (all randomized participants receiving at least one dose of study drug). PK parameters were assessed for MAPT_Rx_ in CSF and plasma. Analyses of PD biomarkers and exploratory and clinical endpoints were summarized according to treatment group, and MAPT_Rx_ -treated groups were compared with the pooled placebo group. While there is no statistical rationale for the sample size, it has been selected on the basis of prior experience with generation 2.0 ASOs^31^ given by IT bolus injection to ensure that the safety, tolerability, PK and exploratory pharmacodynamics will be adequately assessed while minimizing unnecessary patient exposure. Statistical analyses were performed using Statistical Analysis System (SAS) Version 9.4.

### Reporting summary

Further information on research design is available in the Nature Portfolio Reporting Summary linked to this article.

## Online content

Any methods, additional references, Nature Portfolio reporting summaries, source data, extended data, supplementary information, acknowledgements, peer review information; details of author contributions and competing interests; and statements of data and code availability are available at 10.1038/s41591-023-02326-3.

### Supplementary information

Supplementary Information Supplementary clinical study information.

Reporting Summary

## Figures and Tables

**Extended Data Table 1:**
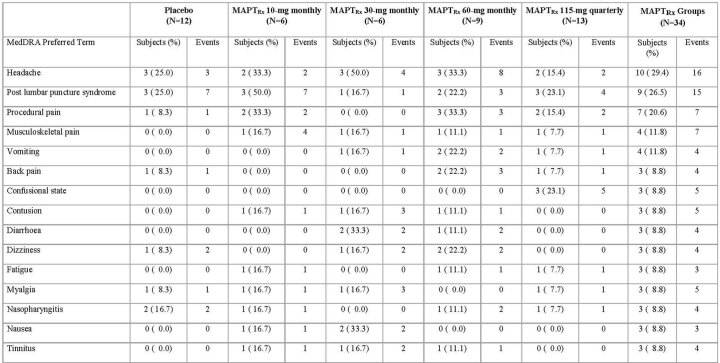
Adverse Events Reported in at Least Three Patients* Receiving MAPT_Rx_ According to Treatment Group

*Patients reporting more than one adverse event were counted only once for the incidence using the most severe grade and, if there was a missing severity for the same subject, then the non-missing severity, if available, was chosen for the same subject.

**All headache events including those related to lumbar puncture procedure.

**Extended Data Table 2:**
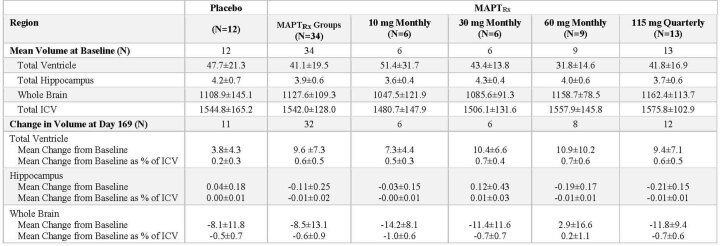
Ventricle, Hippocampus and Whole Brain Volumes*

* Volume measured in cm^3^. Plus–minus values are means ± SD.

ICV: Intracranial Volume (cm^3^)

## Data Availability

To request access to data, please visit Vivli. The individual participant data collected during the trial and that support the research proposal will be available to qualified scientific researchers, in accordance with Biogen’s Clinical Trial Transparency and Data Sharing Policy on www.biogentrialtransparency.com. Data requests are initially reviewed by Vivli and Biogen for completeness and other parameters (relating to scope and meeting sponsor policies) and are then reviewed by an Independent Review Panel. Deidentified data, study protocol and documents will be shared under agreements that further protect against participant reidentification, and data are provided in a secure research environment further protecting participant privacy.
