# Author Correction: Tau-targeting antisense oligonucleotide MAPT_Rx_ in mild Alzheimer’s disease: a phase 1b, randomized, placebo-controlled trial

**DOI:** 10.1038/s41591-023-02639-3

**Published:** 2023-10-16

**Authors:** Catherine J. Mummery, Anne Börjesson-Hanson, Daniel J. Blackburn, Everard G. B. Vijverberg, Peter Paul De Deyn, Simon Ducharme, Michael Jonsson, Anja Schneider, Juha O. Rinne, Albert C. Ludolph, Ralf Bodenschatz, Holly Kordasiewicz, Eric E. Swayze, Bethany Fitzsimmons, Laurence Mignon, Katrina M. Moore, Chris Yun, Tiffany Baumann, Dan Li, Daniel A. Norris, Rebecca Crean, Danielle L. Graham, Ellen Huang, Elena Ratti, C. Frank Bennett, Candice Junge, Roger M. Lane

**Affiliations:** 1 Dementia Research Centre, National Hospital for Neurology and Neurosurgery, University College London, London, UK; 2 Karolinska University Hospital, ME Aging, Stockholm, Sweden; 3 Sheffield Teaching Hospital NHS Foundation Trust, NIHR Sheffield Clinical Research Facility and NIHR Sheffield Biomedical Research Centre, Royal Hallamshire Hospital, Sheffield, UK; 4 Alzheimer Center Amsterdam, Department of Neurology, Amsterdam Neuroscience, Vrije Universiteit Amsterdam, Amsterdam UMC, Amsterdam, the Netherlands; 5 University Medical Center Groningen / RUG, Alzheimer Center Groningen, Groningen, the Netherlands; 6 Douglas Mental Health University Institute and McConnell Brain Imaging Centre of the Montreal Neurological Institute, McGill University, Montreal, Quebec Canada; 7 Memory Clinic, Psychiatry - Cognition and Geriatric Psychiatry, Sahlgrenska University Hospital, Gothenburg/Molndal, Sweden; 8 German Center for Neurodegenerative Diseases, DZNE, and Department of Neurodegenerative Diseases and Geriatric Psychiatry, University Hospital Bonn, Bonn, Germany; 9 CRST Oy; Turku PET Centre University of Turku and Turku University Hospital, Turku, Finland; 10 Department of Neurology University of Ulm and DZNE, Ulm, Germany; 11 Pharmakologisches Studienzentrum Chemnitz GmbH Mittweida, Mittweida, Germany; 12 Ionis Pharmaceuticals, Carlsbad, CA USA; 13 Biogen, Cambridge, MA USA

**Keywords:** Alzheimer's disease, Drug development

Correction to: *Nature Medicine* 10.1038/s41591-023-02326-3. Published online 24 April 2023.

In the version of this article initially published, the affiliation for Daniel Blackburn was incomplete and has been amended to read “Sheffield Teaching Hospital NHS Foundation Trust, NIHR Sheffield Clinical Research Facility and NIHR Sheffield Biomedical Research Centre, Royal Hallamshire Hospital, Sheffield, UK” in the HTML and PDF versions of the article.

