# Supplementary material for: Tau-targeting antisense oligonucleotide MAPTRx in mild Alzheimer’s disease: a phase 1b, randomized, placebo-controlled trial
Source: Nat Med. 2023 Apr 24;29(6):1437–47. doi: 10.1038/s41591-023-02326-3 (PMC10287562; doi:10.1038/s41591-023-02326-3)
Supplement: Supplementary file 1 — Supplementary clinical study information. [file 41591_2023_2326_MOESM1_ESM.pdf]

nature medicine

Article https://doi.org/10.1038/s41591-023-02326-3

Tau-targeting antisense oligonucleotide $MAPT_{Rx}$ in mild Alzheimer's disease: a phase 1b, randomized, placebo-controlled trial

In the format provided by the authors and unedited

Table of Contents

Study Site Team List 2

Ethics Committees Approving Clinical Study 3

Amendments to the Protocol after Commencement of the Study 4

Protocol Eligibility Criteria 5

Table S1: Exploratory assessment values at baseline and changes from baseline to endpoint* 6

Study Site Team List

Dementia Research Centre and Leonard Wolfson Experimental Neurology Centre, National Hospital for Neurology and Neurosurgery, UCL: Catherine Mummery, Filomena Di Bernado, Shazia Begum, Joshua Elflein, Miguel Grilo, Harjit Bhangra, Rosalind Strang

Sheffield Teaching Hospital NHS Foundation Trust Clinical Research Facility, Royal Hallamshire Hospital: Daniel Blackburn, Grace Cole, Alex Radford, Patrick Easton

Montreal Neurological Institute: Simon Ducharme, Angela Genge, Michal Friedman, Salma Khalil, Cyrena Gerardi

Clinical Research Services Turku, CRST Oy: Juha Rinne, Anne Lithonius, Zsofia Lovro

Klinik für Neurologie, Universitätsklinikum Ulm: Albert Ludolph, Christine von Arnim, Therese Grözinger, Carmen Schäfer, Daniela Taranu, Dörthe Polivka

Katholisches Klinikum Bochum gGmbH, Klinik für Neurologie: Siegfried Muhlack, Barbara Kaminski, Daniela Kaminski, Lennard Herrmann

Pharmakologisches Studienzentrum Chemnitz GmbH: Ralf Bodenschatz, Anne Merkel, Caroline Werner

QPS Nederland: Peter Paul de Deyn, Marieke Ettema, Izaak den Daas

Karolinska University Hospital, ME Aging: Anne Börjesson-Hanson, Pia Andersen, Niels Andreasen, Ann-Christine Tysen-Backstrom, Marie Lärksäter, Carolina Hilliä, Karin Nordin, Staffan Rosenborg

Minnesmottagningen, Sahlgrenska Universitetssjukhuset Göteborg/Mölndal: Michael Jonsson, Maria, Berglund, Fredrika Jönsson, Annie Segerbom, Timothy Hadarsson, Samih Almudafar, Dan Curiac, Chrichan Månsson, Sari Huusko, Per Nellgård

Deutsches Zentrum für Neurodegenerative Erkrankungen e. V. (DZNE): Anja Schneider, Guido Hennes, Daniela Krenzel, Bianca Jacobs, Carolin Mikliz, Cornelia McCormick

VUmc, Amsterdam Brain Research Center (BRC): Everard Vijverberg, Philippus Scheltens, Evelien Norbat, Kim Verheul, Dewi Catton, Femke Van Der Linden, Kim Van Geemen, Lisa Van Maanen, Femke Errens

Ethics Committees Approving Clinical Study

Principal Investigator	IRB/Ethic Committee	Reference number
Dr Albert Ludolph	Ethikkommission der Universität Ulm	412/16
Dr Siegfried Muhlack	Ethik-Kommission der Ruhr-Universität Bochum	16-5929
Dr Catherine Mummery	London-Central Research Ethics Committee Manchester HRA Centre	17/LO/0440
Dr Simon Ducharme	MUHC Neurosciences Research Ethics Board	2017-3206
Dr Juha Rinne	National Committee on Medical Research Ethics	73/06.00.01/2017
Dr Ralf Bodenschatz	Ethikkommission der Sächsischen Landesärztekammer	EK-AMG-MCB-155/16-1
Dr Peter Paul de Deyn	Central Committee on Research Involving Human Subjects	NL60032.000.16
Dr Anne Borjesson Hansen	Regionala etikprövningsnämnden i Stockholm Karolinska Institutet i Solna	2017/300-31
Dr Michael Jonsson	Regionala etikprövningsnämnden i Stockholm Karolinska Institutet i Solna	2017/300-31
Dr Daniel Blackburn	London-Central Research Ethics Committee Manchester HRA Centre	17/LO/0440
Dr Anja Schneider	Ethikkommission an der Medizinischen Fakultät der Rheinischen Friedrich-Wilhelms-Universität Bonn	035/18-AMG
Dr Phillipus Scheltens	Central Committee on Research Involving Human Subjects	NL60032.000.16

Amendments to the Protocol after Commencement of the Study

While the study was underway, the inclusion criteria #3 for the Clinical Dementia Rating (CDR) score was amended to include both CDR global score of 1 (mild dementia) and CDR global score of 0.5 (very mild dementia) with a Memory box score of 1 (Amendment 3; May 2018). A subsequent amendment was made to add the Tau-PET ligand, MK6240, as the preferred option for positron emission tomography (PET) imaging. When available at the site Tau-PET should be performed. If Tau-PET was not available at the site, then FDG-PET should have been performed instead. PET analysis will be performed following completion of the open-label long term extension (LTE).

The main amendment (Amendment 5; March 2019) to the original study protocol was the addition of an open-label LTE to the multiple ascending dose (MAD) part of the study. The amended protocol included the MAD as Part 1, and the LTE as Part 2, with the visit numbers restarting at Day -1 in the LTE, Part 2. The amendment specified how patients in the different Cohorts would flow into the LTE, Part 2.

In 2020, a protocol addendum was made to address the impact of the COVID-19 pandemic on the conduct of ISIS 814907-CS1 study. At the time of the addendum, the study had completed enrollment with 46 patients randomized. In MAD Part 1 of the study, 25 patients had completed all visits, and 20 were still in the Treatment or Post-Treatment Periods.

Protocol Eligibility Criteria

In addition to the requirements described in the main report, all participants were judged by the Investigator to be capable of giving informed consent, and all participants provided written consent for participation in the study. All participants were judged by the Investigator as able and willing to meet study requirements, including travel to the study center and ability to undergo all study procedures and to abide by study restrictions.

Exclusion criteria included any condition that would have prevented the patient from participation in writing tasks, MRI or lumbar puncture (LP). Patients deemed by the Investigator to be at significant risk of suicide, major depressive episode, psychosis, confusional state or violent behavior were excluded. Clinically significant laboratory, vital sign and electrocardiogram abnormalities at screening were exclusionary as was a medical history of brain or spinal disease that would be expected to interfere with CSF circulation. Presence of an implanted shunt for the drainage of cerebral spinal fluid (CSF) or implanted central nervous system catheter was not permitted.

Table S1: Exploratory assessment values at baseline and changes from baseline to endpoint*

	Baseline					Change from Baseline to Endpoint*				
	Placebo (N=12)	MAPT$_{Rx}$ 10 mg (N=6)	MAPT$_{Rx}$ 30 mg (N=6)	MAPT$_{Rx}$ 60 mg (N=9)	MAPT$_{Rx}$ 115 mg (N=13)	Placebo (N=12)	MAPT$_{Rx}$ 10 mg (N=6)	MAPT$_{Rx}$ 30 mg (N=6)	MAPT$_{Rx}$ 60 mg (N=9)	MAPT$_{Rx}$ 115 mg (N=13)
**CSF ABeta1-42 (% change from baseline)**										
Mean pg/mL (SD)	700.1±245.2	656.4±176.9	663.5±90.5	759.3±179.1	716.0±182.4	-1.5± 9.2	-14.4 ±5.8	-23.3± 4.1	-22.5±8.8	-10.4 ±17.5
Median	656.5	661.7	655.8	767.3	687.1	-2.6	-13.5	-21.7	-19.6	-12.7
Min, Max	340.4, 1126.0	428.5, 890.4	568.3, 800.7	500.4, 1059.5	470.3, 1039.0	-17.0, 12.7	-23.2, -7.1	-30.5, -20.7	-35.9, -12.2	-35.8, 20.0
**CSF Phospho-Tau (% change from baseline)**										
Mean pg/mL (SD)	38.7±13.0	39.1±13.0	38.6±16.6	39.5±12.6	43.2±15.9	-2.7±11.5	-35.0 ±8.1	-43.8 ±7.2	-52.0 ±7.6	-48.5 ±15.2
Median	39.2	36.5	32.8	36..0	39.4	-1.8	-34.8	-46.2	-51.3	-55.2
Min, Max	20.6, 57.9	26.5, 64.0	23.0, 69.1	19.7, 56.1	24.2, 81.3	-32.2, 7.8	-44.1, -22.5	-51.2, -32.4	-66.8, -44.3	-67.2, -14.2
**CSF Total-Tau to ABeta-42 Ratio (% change from baseline)**										
Mean (SD)	0.6±0.2	0.6±0.2	0.6±0.1	0.5±0.1	0.6±0.2	0.6±11.9	-17.1 ±14.9	-21.3± 9.7	-33.8 ±9.8	-35.5±10.8
Median	0.6	0.6	0.5	0.5	0.6	2.2	-24.2	-21.9	-36.7	-37.2
Min, Max	0.4, 1.0	0.3, 0.8	0.4, 0.8	0.4, 0.8	0.4, 1.2	-24.4, 20.3	-25.7, 9.3	-34.7, -10.6	-44.5, -13.9	-52.8, -17.8
**CSF Total-Tau (% change from baseline)**										
Mean pg/mL (SD)	387.3±120.9	364.6±98.1	386.4±152.3	391.0±111.8	443.4±153.8	-1.3±11.4	-29.5±8.0	-39.7±5.8	-48.8 ±6.9	-41.9 ±15.4
Median	398.7	330.4	331.0	345.1	399.0	-2.1	-30.5	-38.1	-45.9	-47.6
Min, Max	212.0, 566.8	281.1, 556.1	251.8, 659.3	221.1, 537.8	264.0, 811.8	-27.9, 15.9	-35.7, -16.0	-48.4, -32.6	-62.5, -43.3	-60.0, -5.0
**CSF NfL (% change from baseline)**										
Mean pg/mL (SD)	1359.5 (376.5)	1228.5 (387.5)	1501.9 (483.5)	1189.9 (425.2)	1282.9 (381.6)	3.2 (11.1)	15.6 (39.5)	20.7 (15.2)	22.8 (34.6)	8.3 (7.2)
Median	1466.0	1128.3	1314.4	1192.4	1167.6	-0.4	-3.8	20.4	4.7	10.1
Min, Max	581.0, 1850.2	807.0, 1903.7	1111.2, 2391.8	630.8, 1992.8	737.7, 2223.5	-12.2, 22.2	-20.1, 79.6	6.7, 44.3	-13.4, 82.6	-3.1, 19.4
**CSF NRGN (% change from baseline)**										
Mean pg/mL (SD)	475.9 (195.1)	488.7 (219.1)	515.7 (269.3)	570.6 (235.5)	571.976 (264.6)	1.9 (20.5)	-26.0 (12.1)	-25.7 (15.4)	-13.9 (20.4)	7.4 (29.3)
Median	502.3	420.077	449.235	470.174	541.691	1.3	-30.7	-30.0	-14.2	0.3
Min, Max	194.6, 808.4	315.3, 905.4	237.8, 1013.2	302.2, 1017.3	280.0, 1306.9	-38.1, 35.2	-37.7, -6.7	-42.9, -2.2	-43.9, 17.0	-33.6, 76.2
**CSF NfH (Total) (% change from baseline)**										
Mean pg/mL (SD)	1277.2 (579.9)	965.1 (108.3)	1294.2 (702.9)	1258.6 (448.0)	1183.8 (375.3)	0.8 (15.0)	25.8 (52.5)	58.9 (82.1)	31.1 (32.3)	15.3 (15.9)
Median	1184.1	937.1	1146.6	1094.1	1097.4	5.0	18.3	25.4	15.9	17.8
Min, Max	578.7, 2539.6	839.04,1107.6	649.4, 2566.8	747.3, 2064.8	785.4, 1879.3	-31.6, 17.9	-19.9, 114.2	16.4, 205.6	-4.7, 66.4	-12.9, 44.9
**CSF YKL40 (% change from baseline)**										
Mean ng/mL (SD)	221.5 (57.6)	238.8 (93.9)	434.1 (21.6)	251.7 (119.6)	248.7 (70.2)	9.4 (27.9)	-30.3 (13.5)	-30.2 (31.6)	-17.5 (12.5)	-7.1 (11.7)
Median	224.7	236.1	503.2	242.0	235.4	1.4	-38.2	-20.0	-17.7	-10.5
Mix, Max	(139.2, 301.1)	(134.4, 385.4)	(131.2, 662.7)	98.8, 487.1	125.2, 380.1	-10.1, 85.1	-42.0, -10.7	-73.4, -2.1	-34.0, 5.7	-23.3, 13.6
**MRI Diffusion Analysis: Fractional Anisotropy – Fornix**										
Mean (SD)	0.21±0.02	0.19±0.03	0.20±0.03	0.23±0.04	0.20±0.04	0.00±0.02	-0.01±0.00	-0.01±0.01	-0.01±0.01	-0.01±0.01
Median	0.21	0.18	0.21	0.23	0.21	-0.01	-0.01	0.00	-0.01	-0.01
Mix, Max	0.16,0.24	0.17,0.24	0.17,0.24	0.16,0.30	0.14,0.26	-0.01, 0.05	-0.02, -0.01	-0.02, 0.00	-0.03, 0.00	-0.02, 0.01
**MRI Diffusion Analysis: Fractional Anisotropy – Forceps Major**										
Mean (SD)	0.69±0.06	0.62±0.07	0.65±0.03	0.69±0.06	0.67±0.05	0.01±0.03	-0.01±0.02	-0.02±0.04	-0.02±0.02	-0.01±0.01
Median	0.71	0.62	0.66	0.68	0.68	0.00	-0.01	-0.01	-0.02	-0.01
Min, Max	0.59,0.76	0.52,0.68	0.58,0.67	0.59,0.78	0.57,0.75	-0.02,0.09	-0.03, 0.02	-0.09, 0.00	-0.04,0.03	-0.04, 0.03

**MRI Diffusion Analysis: Fractional Anisotropy – Cingulum Hippocampus**										
Mean (SD)	0.40±0.04	0.33±0.03	0.35±0.02	0.39±0.06	0.38±0.03	0.01±0.05	0.03±0.02	0.01±0.01	0.00±0.02	0.01±0.01
Median	0.38	0.33	0.35	0.41	0.38	0.01	0.03	0.00	0.00	0.01
Min, Max	0.36, 0.47	0.29, 0.37	0.33, 0.39	0.30, 0.50	0.34, 0.46	-0.04, 0.15	-0.01, 0.05	0.00, 0.02	-0.02, 0.03	-0.02, 0.03
**MRI: Cortical Thickness – Cortical Gray Matter (mm)**										
Mean (SD)	2.9±0.2	2.8±0.2	2.7±0.2	3.1±0.3	2.9±0.2	0.0±0.1	-0.0±0.1	-0.0±0.1	-0.1±0.2	0.0±0.1
Median	2.9	2.7	2.7	3.1	2.8	0.0	-0.0	-0.0	-0.0	0.0
Min, Max	2.6, 3.2	2.5, 3.0	2.5, 3.2	2.8, 3.6	2.6, 3.3	-0.2, 0.2	-0.1, 0.2	-0.2, 0.0	-0.4, 0.0	-0.1, 0.2
**MRI: White Matter Lesion Analysis (cm^3)**										
Mean (SD)	13.9±10.1	10.4±4.6	13.1±6.2	11.7±7.9	9.0±6.9	2.4±2.8	1.4±1.9	5.9±11.2	5.0±9.1	1.6±1.0
Median	11.9	10.9	11.4	11.5	7.2	1.3	0.7	1.2	3.1	1.9
Min, Max	4.0, 42.0	3.9, 15.9	7.1, 24.8	1.8, 26.1	2.1, 26.2	-0.3, 7.8	-0.2, 4.2	-0.5, 28.5	-2.8, 27.0	-0.1, 2.7
**RBANS Total Scale (absolute change from baseline)**										
Mean (SD)	64.9±10.2	58.8±11.2	69.2±12.1	69.9±9.1	70.9±13.4	1.5±7.3	-2.2±5.6	-10.0±8.3	2.6±9.2	-0.6±7.1
Median	65.0	55.5	70.0	72.0	73.0	1.0	-1.5	-7.0	4.5	-3.0
Min, Max	49.0, 80.0	49.0, 80.0	51.0, 82.0	57.0, 85.0	49.0, 90.0	-9.0, 14.0	-12.0, 5.0	-25.0, -3.0	-14.0, 17.0	-11.0, 11.0
**MMSE Total Score (absolute change from baseline)**										
Mean (SD)	24.2±1.7	21.5±1.6	24.5±1.4	24.6±2.5	23.2±2.5	-2.3±2.5	-2.0±1.8	-1.2±3.3	0.9±1.0	-1.0±2.4
Median	23.5	21.0	25.0	26.0	23.0	-2.0	-2.5	-1.5	1.0	0.0
Min, Max	22.0, 27.0	20.0, 24.0	22.0, 26.0	21.0, 27.0	20.0, 27.0	-6.0, 2.0	-4.0, 1.0	-4.0, 5.0	-1.0, 2.0	-8.0, 1.0
**NPI-Q Total Severity Score (absolute change from baseline)**										
Mean (SD)	3.7±6.7	1.0±1.1	5.2±3.8	1.1±2.0	2.0±1.8	-0.7±1.7	0.3±1.6	-1.2±3.5	0.1±2.4	-0.7±2.2
Median	1.0	1.0	5.0	0.0	1.0	0.0	0.0	-0.5	0.0	-1.0
Min, Max	0.0,19.0	0.0, 3.0	0.0,10.0	0.0, 6.0	0.0, 5.0	-4.0, 1.0	-2.0, 3.0	-8.0, 2.0	-5.0, 3.0	-4.0, 4.0
**FAQ Total Score (absolute change from baseline)**										
Mean (SD)	8.3±7.2	6.3±3.5	14.5±4.4	5.6±2.8	9.3±5.1	2.5±5.3	5.2±6.6	2.2±5.9	0.9±3.7	1.5±3.3
Median	6.5	7.0	14.0	5.0	9.0	0.0	4.0	3.5	-0.5	0.0
Min, Max	1.0, 22.0	1.0, 11.0	9.0, 22.0	2.0, 10.0	1.0, 19.0	-6.0, 12.0	-2.0, 14.0	-9.0, 7.0	-2.0, 8.0	-2.0, 6.0

* For CSF parameters, endpoint is defined as Study Day 141 for this table; for structural imaging parameters (Fractional Anisotropy, Cortical Thickness, White Matter Lesion Analysis), endpoint is defined as Study Day 169; for all other parameters, endpoint is defined as Study Day 169.

Exploratory CSF parameters including neurofilament light (NfL) and heavy (NfH), neurogranin (NRGN) and YKL-40 showed no dose-responsive effects following treatment with $MAPT_{Rx}$.
