# Supplementary material for: Tau-targeting antisense oligonucleotide MAPTRx in mild Alzheimer’s disease: a phase 1b, randomized, placebo-controlled trial
Source: Nat Med. 2023 Apr 24;29(6):1437–47. doi: 10.1038/s41591-023-02326-3 (PMC10287562; doi:10.1038/s41591-023-02326-3)
Supplement: Supplementary file 2 — Reporting Summary [file 41591_2023_2326_MOESM2_ESM.pdf]

nature portfolio

Corresponding author(s): Catherine J. Mummery
Email – cath.mummery@nhs.net

Last updated by author(s): Mar 22, 2023

Reporting Summary

Nature Portfolio wishes to improve the reproducibility of the work that we publish. This form provides structure for consistency and transparency in reporting. For further information on Nature Portfolio policies, see our Editorial Policies and the Editorial Policy Checklist.

Statistics

For all statistical analyses, confirm that the following items are present in the figure legend, table legend, main text, or Methods section.

n/a	Confirmed	
☐	☒	The exact sample size (n) for each experimental group/condition, given as a discrete number and unit of measurement
☒	☐	A statement on whether measurements were taken from distinct samples or whether the same sample was measured repeatedly
☒	☐	The statistical test(s) used AND whether they are one- or two-sided<br>*Only common tests should be described solely by name; describe more complex techniques in the Methods section.*
☐	☒	A description of all covariates tested
☐	☒	A description of any assumptions or corrections, such as tests of normality and adjustment for multiple comparisons
☐	☒	A full description of the statistical parameters including central tendency (e.g. means) or other basic estimates (e.g. regression coefficient) AND variation (e.g. standard deviation) or associated estimates of uncertainty (e.g. confidence intervals)
☒	☐	For null hypothesis testing, the test statistic (e.g. F, t, r) with confidence intervals, effect sizes, degrees of freedom and P value noted<br>*Give P values as exact values whenever suitable.*
☒	☐	For Bayesian analysis, information on the choice of priors and Markov chain Monte Carlo settings
☒	☐	For hierarchical and complex designs, identification of the appropriate level for tests and full reporting of outcomes
☒	☐	Estimates of effect sizes (e.g. Cohen's d, Pearson's r), indicating how they were calculated

Our web collection on statistics for biologists contains articles on many of the points above.

Software and code

Policy information about availability of computer code

Data collection	No software was used.
Data analysis	VivoQuant software was used to measure regional brain volumes. SAS version 9.4 was used for data analysis.

For manuscripts utilizing custom algorithms or software that are central to the research but not yet described in published literature, software must be made available to editors and reviewers. We strongly encourage code deposition in a community repository (e.g. GitHub). See the Nature Portfolio guidelines for submitting code & software for further information.

Data

Policy information about availability of data

All manuscripts must include a data availability statement. This statement should provide the following information, where applicable:

- Accession codes, unique identifiers, or web links for publicly available datasets
- A description of any restrictions on data availability
- For clinical datasets or third party data, please ensure that the statement adheres to our policy

To request access to data, please visit Vivli. The individual participant data collected during the trial and that support the research proposal will be available to qualified scientific researchers, in accordance with Biogen's Clinical Trial Transparency and Data Sharing Policy on www.biogentrialtransparency.com. Data requests are initially reviewed by Vivli and Biogen for completeness and other parameters (relating to scope and meeting sponsor policies) and are then reviewed by an

Independent Review Panel. Deidentified data, study protocol and documents will be shared under agreements that further protect against participant reidentification and data are provided in a secure research environment further protecting participant privacy.

Human research participants

Policy information about studies involving human research participants and Sex and Gender in Research.

Reporting on sex and gender	Participant sex was determined by self-reporting. An equal number of male and female participants were included the study (N=23 each, Table 1).
Population characteristics	Covariate-relevant population characteristics including age, sex, race, and genotype are described in Table 1
Recruitment	Study Investigators identified and recruited participants for the study according the study eligibility criteria. The Sponsor is unaware of any patient selection bias.
Ethics oversight	Principal Investigator, IRB/Ethic Committee and Reference number<br><br>Dr Albert Ludolph Ethikkommission der Universität Ulm 412/16<br>Dr Siegfried Muhlack Ethik-Kommission der Ruhr-Universität Bochum 16-5929<br>Dr Catherine Mummery London-Central Research Ethics Committee<br>Manchester HRA Centre 17/LO/0440<br>Dr Simon Ducharme MUHC Neurosciences Research Ethics Board 2017-3206<br>Dr Juha Rinne National Committee on Medical Research Ethics 73/06.00.01/2017<br>Dr Ralf Bodenschatz Ethikkommission der Sächsischen Landesärztekammer EK-AMG-MCB-155/16-1<br>Dr Peter Paul de Deyn Central Committee on Research Involving Human Subjects NL60032.000.16<br>Dr Anne Borjesson Hansen Regionala etikprövningsnämnden i Stockholm<br>Karolinska Institutet i Solna 2017/300-31<br>Dr Michael Jonsson Regionala etikprövningsnämnden i Stockholm<br>Karolinska Institutet i Solna 2017/300-31<br><br>Dr Daniel Blackburn London-Central Research Ethics Committee<br>Manchester HRA Centre 17/LO/0440<br>Dr Anja Schneider Ethikkommission an der Medizinischen Fakultät der Rheinischen Friedrich-Wilhelms-Universität Bonn 035/18-AMG<br>Dr Phillipus Scheltens Central Committee on Research Involving Human Subjects NL60032.000.16

Note that full information on the approval of the study protocol must also be provided in the manuscript.

Field-specific reporting

Please select the one below that is the best fit for your research. If you are not sure, read the appropriate sections before making your selection.

☒ Life sciences ☐ Behavioural & social sciences ☐ Ecological, evolutionary & environmental sciences

For a reference copy of the document with all sections, see nature.com/documents/nr-reporting-summary-flat.pdf

Life sciences study design

All studies must disclose on these points even when the disclosure is negative.

Sample size	While there is no statistical rationale for the sample size, it has been selected based on prior experience with generation 2.0 ASOs (Tabrizi et. al, 2019) given by IT bolus injection to ensure that the safety, tolerability, PKs, and exploratory pharmacodynamics will be adequately assessed while minimizing unnecessary patient exposure.
Data exclusions	No data was excluded from the analyses
Replication	The study findings have not yet been replicated in other human clinical studies. There is an ongoing Phase 2 study evaluating BIIB080 (MAPTRx) in patients with mild cognitive impairment and mild Alzheimer's disease (NCT05399888).
Randomization	In MAD, Part 1, of the study, a patient was randomized after all Screening assessments have been completed and after the Investigator has verified that the patient is eligible per criteria. No patient may begin treatment prior to randomization and assignment of a unique patient identification number.<br>Eligible patients were randomized centrally by an automated system to receive ISIS 814907 or placebo. Within each cohort, randomization was 3:1 ISIS 814907: placebo
Blinding	The investigators, patients and study center personnel, including the site pharmacist, were blinded

Blinding	to treatment assignment for the duration of the study (i.e., until the end of long-term extension).<br><br>An interim analysis of the multiple ascending dose (MAD) Part 1 to investigate safety, PK, PD and exploratory endpoints was conducted at the end of MAD, Part 1 when the last patient completed the last visit. Unblinded data was evaluated at this analysis. All patients/all data through the end of MAD, Part 1 were used for the analysis with the exception of CSF biomarkers data (through Day 1 pre-dose in LTE, Part 2). The individuals involved in the unblinded interim analysis were identified and documented at the time of unblinded interim analysis according to Ionis standard operation procedure (SOP).

Reporting for specific materials, systems and methods

We require information from authors about some types of materials, experimental systems and methods used in many studies. Here, indicate whether each material, system or method listed is relevant to your study. If you are not sure if a list item applies to your research, read the appropriate section before selecting a response.

Materials & experimental systems

n/a	Involved in the study
☒	☐ Antibodies
☒	☐ Eukaryotic cell lines
☒	☐ Palaeontology and archaeology
☒	☐ Animals and other organisms
☐	☒ Clinical data
☒	☐ Dual use research of concern

Methods

n/a	Involved in the study
☒	☐ ChIP-seq
☒	☐ Flow cytometry
☐	☒ MRI-based neuroimaging

Clinical data

Policy information about clinical studies

All manuscripts should comply with the ICMJE guidelines for publication of clinical research and a completed CONSORT checklist must be included with all submissions.

Clinical trial registration	NCT03186989
Study protocol	The full trial protocol has not been made public. Biogen will provide the study protocol upon approval of a research proposal per the data availability statement.
Data collection	From August 2017 through February 2020, 102 participants were screened for eligibility and 46 underwent randomization according to the protocol. Data was collected at the study sites listed below for MAD Part 1 through September 2020 (study site information is also provided in the supplement). Data were single-entered into the electronic data capture system by the clinical site staff.<br>Dementia Research Centre and Leonard Wolfson Experimental Neurology Centre, National Hospital for Neurology and Neurosurgery, UCL: Catherine Mummery, Filomena Di Bernado, Shazia Begum, Joshua Elflein, Miguel Grilo, Harjit Bhangra, Rosalind Strang<br>Sheffield Teaching Hospital NHS Foundation Trust Clinical Research Facility, Royal Hallamshire Hospital: Daniel Blackburn, Grace Cole, Alex Radford, Patrick Easton<br>Montreal Neurological Institute: Simon Ducharme, Angela Genge, Michal Friedman, Salma Khalil, Cyrena Gerardi<br>Clinical Research Services Turku, CRST Oy: Juha Rinne, Anne Lithonius, Zsofia Lovro<br>Klinik für Neurologie, Universitätsklinikum Ulm: Albert Ludolph, Christine von Arnim, Therese Grözinger, Carmen Schäfer, Daniela Taranu, Dörthe Polivka<br>Katholisches Klinikum Bochum gGmbH, Klinik für Neurologie: Siegfried Muhlack, Barbara Kaminski, Daniela Kaminski, Lennard Herrmann<br>Pharmakologisches Studienzentrum Chemnitz GmbH: Ralf Bodenschatz, Anne Merkel, Caroline Werner<br>QPS Nederland: Peter Paul de Deyn, Marieke Ettema, Izaak den Daas<br>Karolinska University Hospital, ME Aging: Anne Börjesson-Hanson, Pia Andersen, Niels Andreasen, Ann-Christine Tysen-Backstrom, Marie Lärksäter, Carolina Hilliä, Karin Nordin, Staffan Rosenborg<br>Minnesmottagningen, Sahlgrenska Universitetssjukhuset Göteborg/Mölndal: Michael Jonsson, Maria, Berglund, Fredrika Jönsson, Annie Segerbom, Timothy Hadarsson, Samih Almudafar, Dan Curiac, Chrichan Månsson, Sari Huusko, Per Nellgård<br>Deutsches Zentrum für Neurodegenerative Erkrankungen e. V. (DZNE): Anja Schneider, Guido Hennes, Daniela Krenzel, Bianca Jacobs, Carolin Mikliz, Cornelia McCormick<br>VUmc, Amsterdam Brain Research Center (BRC): Everard Vijverberg, Philippus Scheltens, Evelien Norbat, Kim Verheul, Dewi Catton, Femke Van Der Linden, Kim Van Geemen, Lisa Van Maanen, Femke Errens
Outcomes	Safety and PK were the primary and secondary endpoints, respectively, and prospectively defined in the study protocol. Safety data were summarized according to treatment group. Quantitative assessments were summarized using descriptive statistics including number of patients, mean, median, standard deviation, standard error of mean, interquartile range (25th percentile, 75th percentile), and range (minimum, maximum). Qualitative assessments were summarized using frequency counts and percentages. All safety analyses were performed in the safety population (all randomized participants receiving at least one dose of Study Drug). PK parameters were assessed for MAPTRx in CSF and plasma. Analyses of PD biomarkers, exploratory and clinical endpoints were summarized according to treatment group, and MAPTRx-treated groups were compared with the pooled placebo group. Statistical analyses were performed using Statistical Analysis System (SAS) Version 9.4.

Magnetic resonance imaging

Experimental design

Design type	Resting state
Design specifications	We obtained 3-dimensional (3D) T1-weighted structural MRI scans of the head at baseline and Day 169.
Behavioral performance measures	Not applicable

Acquisition

Imaging type(s)	3-dimensional (3D) T1-weighted structural MRI scans
Field strength	1.5 Tesla
Sequence & imaging parameters	T2 FLAIR, GRE T2 star, T2 Fast Spin Echo [FSE]/Turbo Spin Echo [TSE]
Area of acquisition	whole brain

Diffusion MRI ☐ Used ☒ Not used

Preprocessing

Preprocessing software

Analysis was performed with a validated pipeline implemented in VivoQuantTM, which is comprised of preprocessing module and a multi-atlas segmentation module, followed by visual inspection and manual editing if needed. Trained personnel executed the automated preprocessing pipeline for each MRI data set. The preprocessing pipeline consists of the following steps:
1. Resampling to an isotropic voxel size of 1x1x1 mm.
2. Generating a foreground mask to remove the background noise.
3. Denoising with an adaptive non-local mean filter. This step removes noise and improves signal-to-noise ratio while preserving local structure.
4. B1 nonuniformity bias field correction.8 This step removes potential low frequency intensity nonuniformities in the image.
5. Anterior commissure (AC) – posterior commissure (PC) alignment.9 This step provides an initial alignment of the image using automated identification of the AC-PC to improve downstream image registration.
AC-PC alignment will be visually inspected and manually adjusted by the IPS, if needed. If any manual adjustment is performed by the IPS, then a second IPS will QC the alignment to verify.
7. Intensity normalization. This step harmonizes signal intensities within the brain to improve downstream image registration.
8. Cropping. This step automatically removes potential nuisance regions outside the brain, particularly in the neck/shoulders that may impact downstream image registration

Normalization

Multi-atlas segmentation was performed using a pre-labeled reference library. The steps to build and use the reference library are:
1. The reference library images are selected from the Alzheimer’s Disease Neuroimaging Initiative (ADNI) data sets from healthy controls and patients.
Frisoni GB, Jack CR, Jr., Bocchetta M, et al. The EADC-ADNI Harmonized Protocol for manual hippocampal segmentation on magnetic resonance: evidence of validity. Alzheimers Dement. 2015;11(2):111-125.
2. ROI segmentations have been previously performed under the guidance of neuroanatomical experts and according to published protocols.
Avants BB, Tustison NJ, Song G, Cook PA, Klein A, Gee JC. A reproducible evaluation of ANTs similarity metric performance in brain image registration. Neuroimage. 2011;54(3):2033-2044.
3. A subset of reference library data sets and their associated ROIs, typically 50 ± 20 patients, are selected for a specific study to provide a representative cross-section of the prescribed study population in terms of age, and other demographics, where possible.

Normalization template

Reference library images are selected from the Alzheimer’s Disease Neuroimaging Initiative (Frisoni GB, Jack CR, Jr., Bocchetta M, et al. The EADC-ADNI Harmonized Protocol for manual hippocampal segmentation on magnetic resonance: evidence of validity. Alzheimers Dement. 2015;11(2):111-125)

Noise and artifact removal

De-noising was performed according to published algorithms.
Buades A, Coll B, Morel JM. A Review of Image Denoising Algorithms, with a New One. Multiscale Modeling & Simulation. 2005;4(2):490-530.
Tristán-Vega A, García-Pérez V, Aja-Fernández S, Westin C-F. Efficient and robust nonlocal means denoising of MR data based on salient features matching. Computer Methods and Programs in Biomedicine. 2012;105(2):131-144.

Volume censoring

Segmentations of each ROI are generated via implementation of a multi-atlas segmentation (MAS)

Volume censoring

strategy. The MAS module is executed as an automated pipeline within a validated version of the VivoQuant software, followed by visual inspection and manual editing, if needed. The input is the preprocessed T1 MRI. MAS steps include:
1. Each MRI from the reference library along with its associated labels are affine-registered to the target image space. (Wang H, Suh JW, Das SR, Pluta JB, Craige C, Yushkevich PA. Multi-Atlas Segmentation with Joint Label Fusion. IEEE Transactions on Pattern Analysis and Machine Intelligence. 2013;35(3):611-623)
2. For each ROI, a bounding box comprising the union of the corresponding labels from all reference library images in the target image space is generated. This step restricts the registration region to improve registration accuracy and decrease processing time.
3. Regions within the bounding box of the reference MRIs are warped to the target image using deformable registration.
4. A subset of the best reference MRIs is selected based on the normalized mutual information (MI) between the target image and each warped reference MRI within the bounding box. This step excludes poorly registered reference library data sets.
5. The warped ROIs corresponding to the best reference MRIs are fused to complete the segmentation. (Wang H, Suh JW, Das SR, Pluta JB, Craige C, Yushkevich PA. Multi-Atlas Segmentation with Joint Label Fusion. IEEE Transactions on Pattern Analysis and Machine Intelligence. 2013;35(3):611-623)
6. Steps 2 through 5 are repeated for each ROI.
7. All ROIs are visually inspected and manually edited by if needed.
The performance of this tool has been validated using publicly available data and achieved high overlap with the manual segmentation and performed favorably relative to Freesurfer in sensitively quantifying region brain volume changes (Wang X, Ghayoor A, Novicki A, Holmes S, Seibyl J, Hesterman J. Application of a Multi-Atlas
Segmentation Tool to Hippocampus, Ventricle and Whole Brain Segmentation. Alzheimer's & Dementia: The Journal of the Alzheimer's Association. 2017;13(7):P1385-P1386.)

Statistical modeling & inference

Model type and settings

Analysis of covariance (ANCOVA) model was used to compare treatment groups at each post-baseline visit. The nonparametric test, Wilcoxon rank-sum test, was performed instead when significant departures from normality were observed. When ANCOVA was applied, the model included treatment groups as factors and baseline value and baseline age as covariates.

Effect(s) tested

Change from Baseline were compared between each MAPTRx-treated group and pooled placebo using ANCOVA or Wilcoxon rank-sum test, as appropriate.

Specify type of analysis: ☐ Whole brain ☒ ROI-based ☐ Both

Anatomical location(s)

Multi-atlas segmentation was performed using a pre-labeled reference library. The reference library images are selected from the Alzheimer's Disease Neuroimaging Initiative (ADNI) data sets from healthy controls and patients.
Frisoni GB, Jack CR, Jr., Bocchetta M, et al. The EADC-ADNI Harmonized Protocol for manual hippocampal segmentation on magnetic resonance: evidence of validity. Alzheimers Dement. 2015;11(2):111-125.

Statistic type for inference
(See Eklund et al. 2016)

Point estimates, standard error, and 95% confidence intervals of least squared (LS) mean within treatment groups, and these statistics along with p-value for treatment difference between each MAPTRx-treated group and pooled placebo were obtained from ANCOVA model. When Wilcoxon rank-sum test was performed, only p-values were provided.

Correction

Due to exploratory purpose of Phase I study, p-values were not adjusted for multiple comparisons.

Models & analysis

n/a	Involved in the study
☒	☐ Functional and/or effective connectivity
☒	☐ Graph analysis
☒	☐ Multivariate modeling or predictive analysis
